# Evolving Trends in Dental Services in Aging Japan: An Age–Period–Cohort Analysis Using Nationwide Data from Fiscal Years 2016 to 2023

**DOI:** 10.3390/dj14020102

**Published:** 2026-02-11

**Authors:** Asuka Takeda, Katsuo Oshima, Hideki Fukuda

**Affiliations:** 1 Department of Health Crisis Management, National Institute of Public Health, Saitama 351-0197, Japan; 2 Department of Oral Health, The Nippon Dental University, Tokyo 102-8159, Japan; 3 National Institute of Public Health, Saitama 351-0197, Japan

**Keywords:** dental service, oral health, aging society, Japan’s National Database of Health Insurance Claims, age–period–cohort analysis

## Abstract

**Background/Objectives**: Understanding changes in dental service utilization is vital for planning effective oral health strategies in aging societies. In this study, we aimed to elucidate nationwide trends in major dental procedures in Japan from fiscal year (FY) 2016 to FY2023, and to assess the age, period, and cohort effects underlying these trends. **Methods**: Using open data from Japan’s National Database of Health Insurance Claims, five procedure types were analyzed: cavity filling, dental calculus removal, tooth extraction, dental crown procedures, and denture procedures. Descriptive analyses were performed to examine the annual and age-specific changes in the number of procedures per 1000 population. Age–period–cohort (APC) analyses were conducted using Poisson regression with spline functions, applying 10-year age groups. **Results**: From FY2016 to FY2023, restorative and prosthetic procedures, including cavity fillings, crowns, and dentures, demonstrated a steady decline, whereas preventive procedures, such as dental calculus removal increased, particularly among younger age groups. The APC analysis revealed distinct age-, period-, and cohort-related patterns in dental service utilization. Age effects indicated relatively higher rates of prosthetic procedures among older adults, whereas cohort effects suggested generational improvements in oral health. Period effects showed a downward shift beginning in FY2020, temporally aligned with the coronavirus disease pandemic. **Conclusions**: The combined descriptive and APC analyses indicate evolving patterns in dental service utilization in Japan, characterized by increased preventive care among younger generations and persistent age-related differences in prosthetic service use. These findings provide population-based evidence relevant for planning sustainable oral healthcare systems in aging societies.

## 1. Introduction

Acknowledging the worldwide public health burden of major dental diseases and conditions, the World Health Assembly (WHA) adopted resolution WHA74.5 on oral health in May 2021 [1]. This resolution urges the integration of oral health into strategies for addressing noncommunicable diseases (NCDs) and achieving Universal Health Coverage (UHC). In 2023, the World Health Organization published the Global Oral Health Action Plan 2023–2030 (GOHAP) to support the implementation of the WHA74.5 resolution on integrating oral health into NCDs and UHC strategies [2,3]. GOHAP is organized around six strategic objectives and defines 11 global oral health targets, including the ensuring access to essential dental service and monitoring the implementation of national oral health policies, to be attained by 2030 [2].

Recent global demographic shifts have influenced oral healthcare systems [4]. With aging populations and declining birth rates, particularly in developed countries, it has become increasingly important to explore these trends and evaluate future oral health demand [5]. Understanding evolving trends in dental service is essential for evaluating changes in healthcare service delivery and patient preferences [6,7]. Analyses of recent multi-year data provides valuable insights into how societal, demographic, and technological factors shape oral healthcare practices [8]. A tailored approach based on age-specific demand is crucial for formulating effective oral health policies to achieve the targets set by GOHAP. Monitoring these changes will foster the development of comprehensive and effective oral healthcare strategies.

In Japan, several oral health problems remain highly prevalent throughout life, although their patterns differ markedly by age group. Dental caries remains one of the most common oral diseases among children and adolescents, despite a long-term decline in prevalence attributable to school-based oral health programs and widespread fluoride use [9,10,11]. Among working-age adults, periodontal disease is a leading cause of tooth loss and is highly prevalent, reflecting cumulative exposure to behavioral and lifestyle risk factors [12]. Among older adults, tooth loss, edentulism, and the need for prosthetic treatment remain major oral health concerns, even as the number of retained natural teeth has increased in recent decades [13,14]. This demographic shift has resulted in a growing demand for denture-related and crown restoration services, alongside ongoing needs for periodontal maintenance and supportive care to preserve oral function in later life. These age-specific patterns underscore the importance of monitoring dental service utilization not only in aggregate but also across generations, as changes in disease prevalence and functional needs directly influence the structure of oral healthcare demand.

Japan, one of the fastest-aging societies in the world [15], provides a valuable model for other countries by demonstrating how oral healthcare systems can adapt to satisfy the demand of an aging population. The growing older adult population may require more specialized oral healthcare [16,17,18], whereas younger generations may benefit from a stronger emphasis on preventive measures. Moreover, Japan’s National Health Insurance System, which covers dental service, plays a critical role in providing healthcare access. This universal insurance system ascertains that all citizens are covered and provides broad access to medical and dental care. The National Database of Health Insurance Claims and Specific Health Checkups of Japan (NDB) offers a robust source of data for analyzing healthcare trends, including dental procedures [19]. Although several studies have examined dental service trends, most have focused on specific regions, age groups, or single procedure types; comprehensive nationwide analyses integrating age, period, and cohort perspectives remain limited [20,21,22].

In this study, we aimed to perform a comprehensive analysis of nationwide trends in dental service in aging Japan from fiscal year (FY) 2016 to FY2023 using large-scale claims data from the NDB. By examining changes in the frequency of key dental procedures, we aimed to characterize recent temporal patterns and age-related differences in service utilization. These procedure categories were selected because they represent major domains of dental care—preventive, restorative, and prosthetic services—and account for a substantial proportion of reimbursed dental claims in Japan. In addition, their definitions based on reimbursement codes allow consistent identification over time in administrative data.

## 2. Materials and Methods

### 2.1. Study Design and Data Source

This repeated cross-sectional study utilized the NDB open data for FY2016 to FY2023. In Japan, the FY runs from 1 April to 31 March of the following calendar year. The NDB, publicly available on the official website of the Ministry of Health, Labour and Welfare (MHLW) in Japan, contains nationwide claims information for both inpatient and outpatient services [23]. In this study, only the outpatient dental claims data were used. Inpatient dental services were not included, which may have limited the capture of dental procedures provided to hospitalized or medically complex patients. The NDB is a comprehensive administrative database managed by the MHLW, encompassing data on medical and dental procedures, including health checkups. This allows for a large-scale analysis of healthcare utilization and disease prevalence across different demographic groups. Population estimates for 1 October of each year from 2016 to 2023 were obtained from the Statistics Bureau of Japan.

### 2.2. Variables for Dental Procedures and Related Indicators

The annual number of billed dental procedures was used as a proxy for dental procedures. According to a previously published classification [24], dental procedures were categorized into five key types: (1) cavity fillings, (2) dental calculus removal, (3) tooth extractions, (4) dental crown procedures, and (5) denture procedures.

The five selected key types represent the major dental treatments commonly conducted and reimbursed in Japan. These types—cavity fillings, dental calculus removal, tooth extractions, dental crown procedures, and denture procedures—encompass a broad spectrum of clinical activities, ranging from preventive and restorative care to prosthetic treatment. They also account for a substantial proportion of all dental claims in the NDB, rendering them suitable indicators for analyzing trends in dental service provisions. In particular, the selected restorative, prosthetic, and surgical procedures are defined by reimbursement codes that are typically billed once per tooth per diagnosis, allowing consistent interpretation of annual procedure counts across age groups and years.

Although periodontal disease is a prevalent condition in Japan, it was not included as a separate category because its diagnosis in claims data often reflects administrative coding rather than a clinically confirmed disease status, rendering it difficult to accurately identify true cases. Instead, dental calculus removal was included as a proxy procedure, reflecting aspects of periodontal care that could be objectively identified from the reimbursement codes. Certain procedures, such as root canal therapy and other specialized interventions, were intentionally excluded from this analysis. Root canal treatment often involves multiple visits and repeated billing for a single tooth, with the number of procedures varying substantially depending on treatment complexity and clinical course. Including such procedures could lead to overestimation of service volume and reduce comparability over time.

Procedure types were identified using specific reimbursement codes (Table S1).

### 2.3. Statistical Analysis

To adjust for demographic changes over time, the number of dental procedures was calculated per 1000 population. For each FY from FY2016 to FY2023, the number of procedures per 1000 population was calculated by procedure type. Percentage changes in dental procedures per 1000 population were calculated for each FY from FY2017 to FY2023, using FY2016 as the reference year. Furthermore, the annual percentage changes by age group and procedure type were analyzed from FY2017 to FY2023, using FY2016 as the reference year. Age groups were defined as follows: under 10 years, 10–19 years, 20–29 years, 30–39 years, 40–49 years, 50–59 years, 60–69 years, 70–79 years, and 80 years and over.

To investigate temporal patterns in the provision of the five types of dental procedures, an age–period–cohort (APC) analysis was conducted [25]. Annual counts of dental procedures from FY2016 to FY2023 were aggregated by 10-year age groups and stratified by procedure type. The midpoints of each age group were used as continuous age values. The annual counts of dental procedures by procedure type were treated as the outcome variables, and age-specific population estimates were included as an offset term to model the incidence rates using a Poisson framework. APC analyses were conducted using a spline-based model to estimate the effects of age, period, and cohort. Although the intrinsic estimator (IE) approach has been proposed as a solution to the identification problem inherent in APC analyses [26,27,28], the primary objective of this study was to capture temporal trends rather than to make causal inferences. Therefore, the IE approach was not used, and APC effects were interpreted descriptively rather than causally.

The spline-based APC models were fitted using Stata/SE V.18.5 (Stata Corp., College Station, TX, USA). Natural cubic splines were applied to age, period (year of treatment), and cohort (calculated as the period minus age). The reference period and cohort were set to 2016 and 1970, respectively. Moreover, the logarithm of population size was incorporated as an offset variable. This specification allowed modeling of the outcome as procedure rates per capita while accounting for overdispersion and temporal nonlinearity. Each dental procedure type was analyzed separately to allow for a comparison of APC patterns across types. Interaction terms between age and period and between period and cohort were not included in the primary model to maintain model identifiability; however, additional exploratory models were fitted to evaluate potential interactions. Sensitivity analyses were conducted by varying the reference period and cohort years to assess the robustness of the estimated effects.

This study was conducted using publicly available data and did not include any personally identifiable information. Thus, ethical approval from the institutional review board was not required.

## 3. Results

The NDB open data provide nationwide coverage of outpatient dental claims in Japan. To provide demographic context for interpreting these trends, Japan’s total population in 2023 was approximately 124 million. The age distribution was as follows: under 10 years, 7.2%; 10–19 years, 8.6%; 20–29 years, 10.2%; 30–39 years, 10.8%; 40–49 years, 13.6%; 50–59 years, 14.4%; 60–69 years, 11.9%; 70–79 years, 13.1%; and 80 years and over, 10.1%.

**Figure 1:**
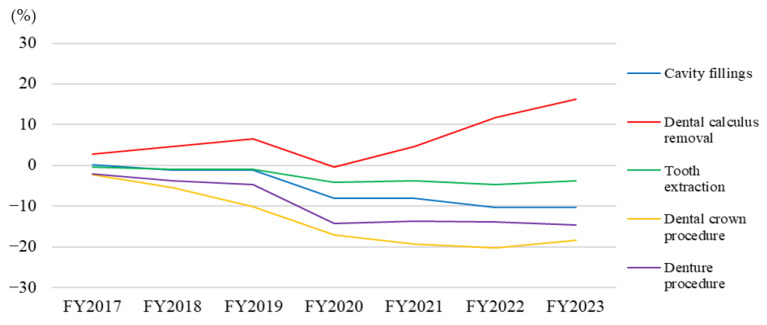
Percentage change in dental procedures per 1000 population, FY2017–FY2023 (baseline: FY2016). The x-axis represents the fiscal year, and the y-axis represents the percent change relative to FY2016. Abbreviations: FY, fiscal year.

As presented in Table 1 and Figure 1, the number of cavity fillings per 1000 population consistently declined throughout the study period (FY2016–FY2023), with all annual changes being calculated relative to FY2016. Procedures for dental calculus removal increased steadily, demonstrating a 16.3% increase by FY2023 compared with that in FY2016. The tooth extraction exhibited a gradual downward trend. Dental crown procedures demonstrated the most substantial decline among all procedure types, with a peak reduction of 20.2% observed in FY2022 compared with that in FY2016. Denture procedures also gradually declined over the study period.

**Figure 2:**
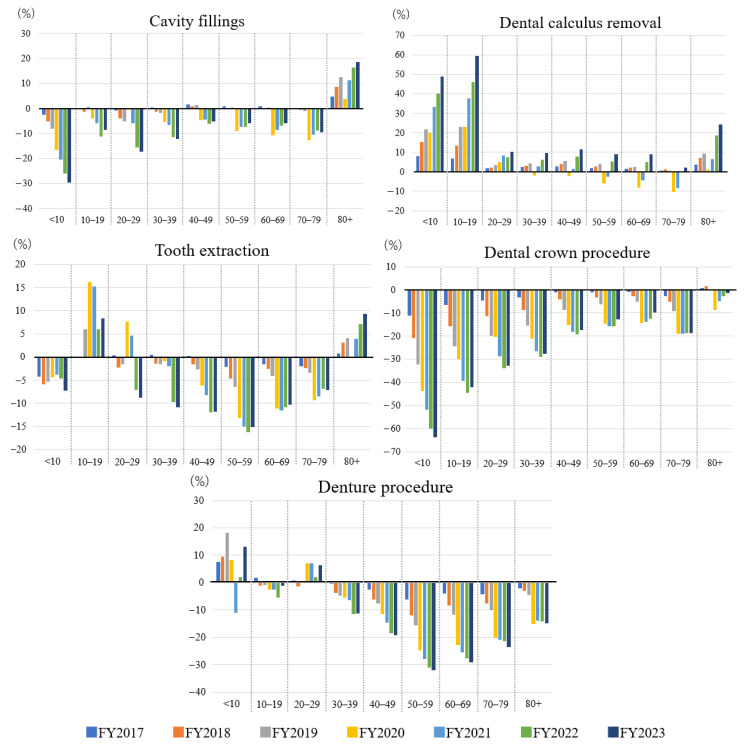
Percentage change in dental procedures per 1000 population by age group and procedure type, FY2017–FY2023 (baseline: FY2016). The x-axis represents the age group, and the y-axis represents the percent change relative to FY2016. Abbreviations: FY, fiscal year.

As shown in Figure 2, cavity-filling procedures declined across all age groups younger than 80 years, with changes calculated relative to that in FY2016. The number of dental calculus removal procedures increased markedly in certain age groups. Particularly, by FY2023, the number of procedures had increased by 48.9% among children under 10 years and by 59.6% among those aged 10–19 years compared with those in FY2016. An increase was also noted in individuals aged 80 years and over. Tooth extraction declined substantially among individuals aged 30 to 59 years. Dental crown procedures showed pronounced year-to-year decreases among individuals aged 30 years and older, with especially steep reductions in younger age groups. By FY2023, compared with that in FY2016, the number of crown procedures had declined by 42.1%, 32.7%, and 27.6% in the 10–19, 20–29, and 30–39-year age groups, respectively. Denture-related procedures exhibited a modest but consistent decline across most age groups.

**Figure 3:**
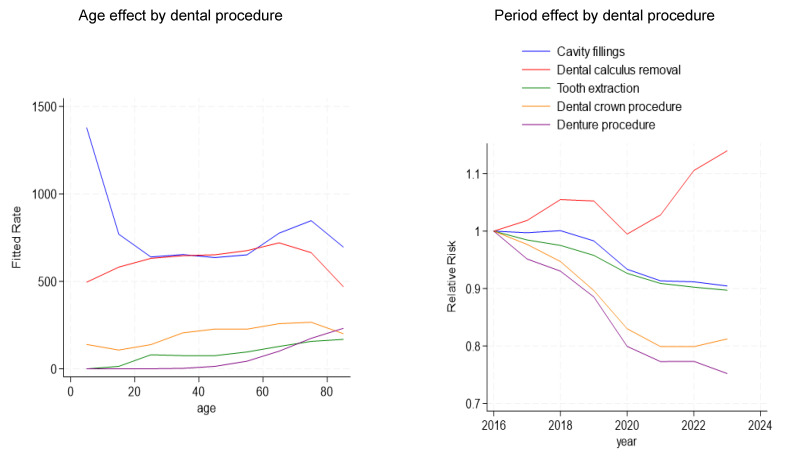
Estimated age and period effects from the age–period–cohort model.

The APC models were used to analyze the five key dental procedure types (Figure 3). All models were fitted using Poisson regression with spline-based parameterization, with the population size included as an offset. Age effects from the APC models represent relative rates across age groups, whereas the descriptive analyses reflect absolute changes in procedure volumes over time. The cohort effect visualization was not included in the main figures due to their interpretational sensitivity; however, full cohort effect estimates for all procedures are summarized in Table S2. For cavity fillings, age effects demonstrated moderate variation across age groups, the period effect indicated a minimal declining trend over time, and the cohort effect showed an increasing risk among younger cohorts. During dental calculus removal, age effects steadily increased with age. Period effects revealed a slight overall decrease, whereas cohort effects declined in recent cohorts. For tooth extraction, pronounced age effects were noted in the middle-aged groups, with decreasing period effects and modest fluctuations across the cohorts. For dental crown procedures, both age and cohort effects were strongly positive, particularly among older individuals and recent birth cohorts. A notable downward drift in the period effect was also noted. In denture procedures, age effects sharply increased with age, period effects showed a consistent decline, and cohort effects demonstrated a downward trend among more recent cohorts. Thus, although the overall number of denture procedures declined during the study period, the APC age effects indicate persistently higher relative utilization among older age groups. All models demonstrated good convergence and indicating that age-, period-, and cohort-related patterns were evident in dental-service utilization.

## 4. Discussion

This nationwide analysis of dental procedure trends from FY2016 to FY2023 provides important insights into how Japan’s oral healthcare system has evolved during a period of demographic transition and external stressors, such as the coronavirus disease (COVID-19) pandemic. Overall, both descriptive and APC model analyses showed consistent declines in restorative and prosthetic procedures alongside increases in preventive services, such as dental calculus removal. These patterns are consistent with a gradual shift in dental service utilization in Japan, from treatment-oriented care toward prevention-oriented oral health management.

The age and cohort effects noted in the APC models reflected the life course and generational differences in oral health needs. The marked increase in denture and crown restorations among older adults corresponds to Japan’s rapidly aging demographic structure, characterized by a longer life expectancy and an increasing number of retained teeth in later life, which together have heightened the demand for prosthetic care [29,30]. Although the absolute number of denture and crown procedures declined over time in the descriptive analyses, the APC age effects indicated higher relative rates among older adults. Therefore, the APC age effect reflects age-related differences rather than an increase in the absolute volume of procedures. Conversely, the pronounced decline in cavity fillings among younger cohorts likely reflects the cumulative impact of long-standing preventive measures, including school-based dental health education and community fluoride mouth-rinsing programs [9,10]. A previous study reported a sustained decline in the prevalence of dental caries among Japanese children and adolescents, indicating that improvements in early life oral health behaviors translate into reduced restorative needs in adulthood [31].

Regarding the period effect, the distinct downward shift starting in FY2020 coincided with the onset of the COVID-19 pandemic. This decline was particularly evident in procedures involving close patient contact and aerosol generation [32,33]. The temporary reduction in dental calculus removal procedures likely reflects the infection prevention measures implemented during the early phase of the pandemic, when dental scaling was recognized as a high-risk aerosol-generating procedure. During this period, the overall utilization of dental care services in Japan substantially decreased [34,35]. Consistent with the results of a previous study [36], these findings indicate that pandemic-related restrictions and voluntary avoidance of non-urgent dental care lead to a temporary reduction in service volumes. Although the number of procedures gradually recovered thereafter, the sustained downward trend in the period effect suggests that the pandemic may have contributed to changes in dental practice patterns, such as possible changes in infection control protocols or patient care-seeking behavior in self-care and preventive behaviors, even within the constraints of Japan’s insurance-based dental system, where remote dental consultations remained uncommon. In addition, the magnitude and persistence of the COVID-19-related period effects appeared to vary across procedure types. Preventive procedures, such as dental calculus removal, showed a relatively rapid recovery following the initial decline, whereas restorative and prosthetic procedures exhibited more prolonged reductions. This heterogeneity may reflect differences in perceived urgency, aerosol generation risk, and clinical prioritization during and after the pandemic, as well as changes in patient care-seeking behavior [36]. Such procedure-specific responses suggest that the pandemic influenced not only the overall volume of dental services but also the composition of care delivered within Japan’s dental healthcare system.

A comparison between the descriptive trends and APC model estimates further clarified the underlying mechanisms of change. For example, the steady decline in crown restorations noted in the raw data was partly attributable to generational improvements in oral health rather than a uniform time effect. Similarly, the increase in calculus removal among children and adolescents might reflect cohort-related differences in preventive orientation, potentially influenced by long-standing school-based programs. These distinctions underscore the analytical value of APC modeling for identifying long-term structural changes that may otherwise be obscured in descriptive analyses [25]. It should be noted that the interpretation of cohort effects in this study is constrained by the relatively short observation window (FY2016–FY2023). Although the APC models captured generational differences in dental service utilization, particularly among younger cohorts, these estimates primarily reflect early-to-mid life stages rather than complete cohort trajectories. Consequently, the observed cohort patterns should be interpreted as indicative of emerging generational trends rather than definitive lifetime effects. Longer observation periods will be required to fully assess how early-life improvements in oral health translate into dental care needs across older ages.

Japan’s experience offers important lessons for other countries experiencing demographic aging and seeking to strengthen their preventive oral healthcare systems. As one of the world’s most rapidly aging societies, Japan has demonstrated how oral healthcare demand can shift from curative to preventive and supportive care across generations. This trend underscores the need for oral healthcare systems to balance investments in geriatric dental services and population-level prevention strategies. For countries undergoing the early stages of population aging, Japan’s trajectory offers a reference model for developing adaptive, resilient oral healthcare frameworks capable of sustaining oral health across the life course. The observed shifts also reinforce the importance of integrating oral health into broader health promotion and chronic disease prevention policies in line with GOHAP [2].

The extent to which these findings can be generalized to other national contexts depends on differences in oral healthcare systems, financing structures, and the availability of population-based preventive programs. Japan’s National Health Insurance System, which provides broad access to dental service with standardized reimbursement, may moderate socioeconomic disparities in service utilization compared with countries relying more heavily on private insurance or out-of-pocket payments [37,38]. Nevertheless, the observed age, period, and cohort patterns—particularly the generational decline in restorative needs and the vulnerability of service utilization to external shocks such as pandemics—are likely relevant to other aging societies [39,40]. Similar age- and cohort-related declines in restorative dental needs have also been reported in other high-income countries undergoing demographic aging, although the magnitude and timing of these changes vary depending on healthcare systems and preventive policies. These cross-national differences provide important context for interpreting the present findings and underscore the contribution of this study to the international literature on oral healthcare system adaptation in aging societies [41]. While the absolute levels of service use may differ, the underlying temporal dynamics identified in this study may offer transferable insights for countries seeking to adapt their oral healthcare systems to demographic aging.

From a clinical perspective, the age- and cohort-specific utilization patterns observed in this study indicate differing dental service needs across the life course. Lower utilization of restorative procedures among younger generations suggests a reduced burden of restorative treatment, whereas the persistently higher relative utilization of prosthetic procedures among older adults highlights the continued need for sufficient capacity in geriatric dental care. From a public health perspective, these findings underscore the importance of aligning oral health policies and service provision with empirically observed utilization trends, including maintaining access to essential dental services for older populations and ensuring system-level preparedness to sustain care during public health emergencies. Future research using longer observation periods and longitudinal or linked datasets would help clarify cohort trajectories and underlying mechanisms.

In summary, the integration of descriptive and APC analyses suggests that Japan’s dental healthcare is entering a transitional phase characterized by generational gains in oral health, shifts in service utilization, and external pressures, including the COVID-19 pandemic. Understanding these dynamics provides not only a domestic basis for planning future oral health policies but also a valuable reference for global efforts to build resilient, preventive, and equitable oral healthcare systems.

This study has some limitations. First, the observation period was relatively short (FY2016–FY2023), limiting the ability to capture long-term changes in dental service utilization, particularly those that may occur gradually over decades. This limitation arises because publicly available NDB open data are currently released only for FY2016 through FY2023, making it impossible to analyze trends beyond this period. Second, the analyses were based on the insurance reimbursement codes required under Japan’s national health insurance system. Accordingly, the recorded procedures may not fully capture the true clinical content or complexity of dental care. Third, self-financed dental procedures were not included in the NDB and were, therefore, excluded from this study. Fourth, although the APC modeling approach provides valuable insights into temporal dynamics, it remains an observational analysis and cannot establish causality. Moreover, APC analysis is sensitive to model specifications (e.g., spline selection and binning of age groups) and potential unmeasured confounding factors [42]. Finally, due to NDB’s open data anonymization and aggregation, individual-level factors, such as socioeconomic status or oral health behaviors, could not be directly evaluated.

## 5. Conclusions

This study described nationwide trends in dental service utilization in Japan between FY2016 and FY2023. The integration of descriptive and APC model analyses demonstrated that declines in restorative and prosthetic procedures alongside increased utilization of preventive services, particularly among younger generations, as well as persistent age-related differences in prosthetic service use. These patterns appear to reflect generational improvements in oral health and changes in service utilization patterns associated with demographic aging and external stressors, such as the COVID-19 pandemic. Future research using longitudinal or linked datasets is required to validate these findings. Rather than indicating a definitive shift in the orientation of dental care delivery, these findings suggest evolving utilization patterns that are increasingly characterized by greater use of preventive services among younger generations and continued demand for supportive and prosthetic care in older populations. The findings can offer valuable insights for countries facing population aging and seeking to develop sustainable oral healthcare systems as well as for other countries facing similar demographic shifts, emphasizing the need to integrate oral health into broader health promotion and chronic disease prevention frameworks. Strengthening resilience of oral healthcare systems is essential for sustaining oral health across the life course, particularly in aging societies.

## Figures and Tables

**Table 1:** Number of dental procedures per 1000 population by procedure type, FY2016–FY2023.

Dental Procedure	Number of Dental Procedures per 1000 Population							
	FY2016	FY2017	FY2018	FY2019	FY2020	FY2021	FY2022	FY2023
Cavity fillings	756.4	758.2	748.0	747.1	695.8	694.9	678.6	678.3
Dental calculus removal	614.0	630.7	642.6	654.4	611.6	642.7	686.6	714.1
Tooth extraction	89.6	89.2	88.8	88.7	86.0	86.3	85.5	86.2
Dental crown procedure	197.6	193.2	186.7	177.5	163.7	159.3	157.7	161.1
Denture procedure	59.7	58.4	57.4	56.9	51.2	51.5	51.4	51.0

Abbreviations: FY, fiscal year.

## Data Availability

The data presented in this study are openly available in [NDB open data] at [https://www.mhlw.go.jp/ndb/opendatasite/] (accessed on 5 February 2026), reference number [23].

## Supplementary Materials

The following supporting information can be downloaded at: https://www.mdpi.com/article/10.3390/dj14020102/s1, Table S1: Insurance claims codes assigned to the five key types of dental procedures. Table S2: Estimated cohort effects from APC model for all procedure types.

## Abbreviations

The following abbreviations are used in this manuscript:

APC	Age–period–cohort
FY	Fiscal year
GOHAP	Global Oral Health Action Plan
IE	Intrinsic estimator
MHLW	Ministry of Health, Labour and Welfare (Japan)
NCDs	Noncommunicable diseases
NDB	National Database of Health Insurance Claims and Specific Health Checkups of Japan
UHC	Universal Health Coverage
WHA	World Health Assembly
