# Supplementary material for: Evolving Trends in Dental Services in Aging Japan: An Age–Period–Cohort Analysis Using Nationwide Data from Fiscal Years 2016 to 2023
Source: Dent J (Basel). 2026 Feb 11;14(2):102. doi: 10.3390/dj14020102 (PMC12939752; doi:10.3390/dj14020102)
Supplement: Supplementary file 1 [file dentistry-14-00102-s001.zip › dentistry-4079775-supplementary.pdf]

**Table. S1 Insurance claim codes assigned to five key type of dental procedures**

| |
|---|
| (1) cavity fillings [313000610 cavity preparation for full-metal crown, 313000710 cavity preparation for non-metal crown, 313000810 cavity preparation for deciduous tooth, 313001210 cavity preparation (simple), 313001310 cavity preparation (complex), 313002010 cavity filling (resin), 313002210 cavity filling (metal)] |
| (2) dental calculus removals [304000410 basic periodontal examination for dental calculus removal (1–9), 304000510 basic periodontal examination for dental calculus removal (10–19), 304000610 basic periodontal examination for dental calculus removal (20–), 304001610 periodontal examination for mixed dentition (for children)] |
| (3) tooth extractions (excluding extraction deciduous tooth or wisdom tooth) [310000210 tooth extraction (front tooth), 310000310 tooth extraction (molar)] |
| (4) dental crown procedures [313024110 mounted (dental crown procedures (per tooth))] |
| (5) denture procedures [313005310 mounted (prosthodontics (per mounting) (plate denture (few missing teeth))), 313005410 mounted (prosthodontics (per mounting) (plate denture (several missing teeth)))], [313005510 mounted (prosthodontics (per mounting) (plate denture (complete denture))] |

**Table. S2 Estimated cohort effects from APC model for all procedure types.**

| Cohort (birth year) | Cavity fillings (Est.; 95%CI) | Dental calculus removal (Est.; 95%CI) | Tooth extraction (Est.; 95%CI) | Dental crown procedure (Est.; 95%CI) | Denture procedure (Est.; 95%CI) |
|---|---|---|---|---|---|
| 1930–1939 | 0.28 (0.28, 0.28) | -0.14 (-0.14, -0.14) | 0.09 (0.06, 0.12) | 0.54 (0.54, 0.55) | -0.17 (-0.27, -0.06) |
| 1940–1949 | -0.18 (-0.18, -0.18) | 0.18 (0.18, 0.18) | -0.01 (-0.04, 0.02) | -0.50 (-0.50, -0.49) | 0.21 (0.11, 0.32) |
| 1950–1959 | -0.20 (-0.20, -0.20) | 0.13 (0.13, 0.13) | 0.01 (-0.02, 0.04) | -0.56 (-0.57, -0.55) | 0.26 (0.16, 0.36) |
| 1960–1969 | -0.20 (-0.20, -0.20) | 0.14 (0.14, 0.15) | 0.04 (0.02, 0.07) | -0.54 (-0.55, -0.53) | 0.27 (0.17, 0.37) |
| 1970–1979 | -0.19 (-0.19, -0.19) | 0.14 (0.14, 0.14) | 0.05 (0.02, 0.07) | -0.50 (-0.51, -0.49) | 0.20 (0.09, 0.30) |
| 1980–1989 | -0.14 (-0.14, -0.14) | 0.16 (0.15, 0.16) | 0.06 (0.03, 0.09) | -0.43 (-0.44, -0.43) | 0.15 (0.04, 0.25) |
| 1990–1999 | -0.09 (-0.09, -0.08) | 0.17 (0.16, 0.17) | 0.06 (0.03, 0.09) | -0.39 (-0.39, -0.38) | 0.04 (-0.07, 0.14) |
| 2000–2009 | -0.17 (-0.17, -0.17) | -0.06 (-0.06, -0.06) | -0.06 (-0.08, -0.03) | -0.32 (-0.33, -0.32) | 0.07 (-0.04, 0.19) |

Abbreviations: Est—Estimate, 95%CI—95% Confidence Interval.
